# Supplementary material for: Effects of a sport-based positive youth development program on youth life skills and entrepreneurial mindsets
Source: PLoS One. 2022 Feb 4;17(2):e0261809. doi: 10.1371/journal.pone.0261809 (PMC8815907; doi:10.1371/journal.pone.0261809)
Supplement: S1 File — (DOCX) [file pone.0261809.s001.docx]

Datafile. https://doi.org/10.6084/m9.figshare.17125589
