# Supplementary material for: Effects of a sport-based positive youth development program on youth life skills and entrepreneurial mindsets
Source: PLoS One. 2022 Feb 4;17(2):e0261809. doi: 10.1371/journal.pone.0261809 (PMC8815907; doi:10.1371/journal.pone.0261809)
Supplement: Attachment — Submitted filename: Response to Reviewers.docx [file pone.0261809.s002.docx]

**Manuscript title**: Effects of a Sport-Based Positive Youth Development Program on Youth Life skills and

Entrepreneurial Mindsets

Response to Reviewers

| **Comments** | **Authors’ response** |
| --- | --- |
|  |  |
| Please find below our response to reviewers comments. We greatly appreciate the feedback and believe that it has strengthened our manuscript. | |
| **All page numbers regarding Reviewer’s comments refer to the original version. Although the Reviewer’s comments are not numbered, we assigned numbers to them to make it easier to find their location.** | **All page numbers regarding Author’s response refer to the new, revised version** |
| **Reviewer_1’s technical comments #1**  Please ensure that your manuscript meets PLOS ONE's style requirements, including those for file naming. | **Authors’ Response:** The manuscript has been revised to meet PLOS ONE's style of requirements.   - 3^rd^ level headings - Only capitalizing the first letter of the first word of headings - Figures and tables (labeling, citing and location) - The figure caption placed in the text, but the figure itself is submitted as a separate tiff file. |
| **Reviewer_1 technical Comment #2**  Please remove any funding-related text from the manuscript and let us know how you would like to update your Funding Statement. | **Authors’ Response**:  The financial disclosure and acknowledgement have been deleted from the manuscript and included in the cover letter. |
| **Reviewer_1 Comment #3**  The introduction is quite long and might benefit from streamlining. In the beginning, the rationale for the study is not stated very clearly and I think this could also benefit from presenting the arguments in a more concise manner. | **Authors’ Response**:  The introduction section has been revised. It is short and succinct. |
| **Reviewer_1 Comment #4**  There are no specific hypotheses presented, in particular not regarding the subscales of the life skills measure, which is in contrast to what is presented in the results section. If the main goal is, in fact, to study the effects on the subscales, this should be argued in the introduction and specific hypotheses should be put forward. | **Authors’ Response**:  We have stated two hypotheses that are in line with the study's target outcomes and data analyses. We believe the change enhances clarity. In the revision we offer a rationale for studying life skills and refer to some of the key life skills were assessed in this study. For the sake of brevity, we could not discuss each of the specific life-skills in the introduction. |
| **Reviewer_1 Comment #5**  The age range from 12 to 20 seems quite wide. What were the reasons for this wide age range and what are potential consequences? | **Authors’ Response**:  Our target population was middle school youth and the typical age range for this school level across the three countries is 12-16 years old. However, it is not unusual for youth in some communities to start school late or experience other delays. This explains the wide age range. The primary goal of our sampling frame was to select sports in public schools that will lead to balance by gender and school level, with school level serving as a proxy for age range. To ensure all possible youth in this cohort were included in the study, we decided not to exclude youth who fell outside the typical age range. We tested for age differences in our outcome variables and found no significant differences at pretest and posttest. Therefore, age had no significant effects in our target outcomes. **A statement on how we addressed potential age effects is provided in the preliminary analysis section.** |
| **Reviewer_1 Comment #6**  How were participants recruited? | **Authors’ Response:**  The research team developed recruitment guidelines that were standard across the three participating countries. These guidelines were used to develop a list of potential participating schools in collaboration secondary school sport association in the participating countries. Factors such as gender balance, type of sport, age range, and proximity to the study cite were considered in developing the list of participating schools. There were some logistical challenges in the actual recruitment process, that included lack of access to some schools and parents’ willingness to let their children do overnight camps. |
| **Reviewer_1 Comment #7**  It seems that the numbers of participants (in particular those of the dropouts) across the subsamples do not add up to the total sample. Please check these numbers. It would also be helpful to describe how missing data was handled. | **Authors’ Response**:  We have corrected the numbers on the table to reflect the subsamples. Thank you for picking this error. Less than 1% of data was missing and missing values were considered to be missing completely at random and to have negligible influence on the findings. Therefore, pairwise deletion was allowed to handle all missing values during data analyses. We added this information to the data analyses section. |
| **Reviewer_1 Comment #8**  On p. 8, it is described that intervention and control group were "very similar" on several variables. This should also be tested. | **Authors’ Response:**  We tested for these differences in our preliminary analyses and found no significant differences. We have improved the reporting to make sure it specifies the variables for which we tested for these differences. |
| **Reviewer_1 Comment #9**  There is conflicting information provided about how participants were assigned to the intervention or the control group (randomly vs. matched for gender and school level). How was this done exactly? | **Authors’ Response:**  We have addressed this query under procedure. The age level matching was achieved by recruiting participants from middle schools across the three countries while gender parity was used in the selection and recruitment of participants as well as assignment to the experimental and control group. We regret that the verbiage created some confusion. |
| **Reviewer_1 Comment #10**  Did I understand it correctly that all participants were active athletes? To what extend does this influence which conclusions can be drawn from the study? | Yes, they all were athletes. We concluded based on pre to posttest improvements in life skills for both the intervention and control group that participation in sport is likely to nurture life skills development. This means acquisition of such skills may happen even if they are not intentionally taught or the goal of a program. The same cannot be said about more specialized skill sets such as entrepreneurial mindsets. Significant changes to entrepreneurial mindsets only happened with the experimental group which received specific training on these skills. We have mentioned this in the conclusion. |
| **Reviewer_1 Comment #11**  Did participants have to pay for camp participation or were there any incentives for participating in the study? | **Authors’ Response:**  Camp related costs, that included housing, meals, and transportation from home to the venues and back were fully covered by the program. No other incentives were given. |
| **Reviewer_1 Comment #12**  Were there any considerations regarding statistical power or how was the sample size determined? | We have revised the Procedure to address this. Sample size estimation was done following Cohen (1992) statistical power analysis. Assuming a small effect size (0.2) from the intervention program at α = 0.05 and a power of 0.80, we estimated that we will need at least 75 participants in the experimental and control group. We therefore recruited 50 middle school athletes from each country and assigned half to the experimental group and the remaining half to the control, ensuring gender balance in the two groups. |
| **Reviewer_1 Comment #13**  In the measure section, it is stated that PYD was assessed. In my view, it would be more accurate to refer to life skills here as this was actually assessed. | **Authors’ Response:**  We have made corrections to reflect that indeed it is life skills that we assessed because PYD is the framework that guided our study. |
| **Reviewer_1 Comment #14**  The low subscale reliabilities for the GET2 should nonetheless be reported. Are they in line with previous literature on the measure, or could they also indicate poor data quality? | **Authors’ Response:**  The GET2 has been widely used and reported in the entrepreneurship literature and has consistently reported high reliability scores for the overall instrument (see added citations in our revised manuscript). In our study, we also found high Cronbach’s alpha scores for the GET2 in both the pre-test and post-test (.83 & .86 respectively). It is less common in the literature to use and report the subscales that can be used with the GET2.  It was, therefore, always our intention to use ONLY the overall GET2 scores.  Although we reported the subscales for the GET2 in our original manuscript, it was never our intention to use them as part of this study, and the Reviewer’s comment helped us to see our reference to sub-scales was unnecessary.  In the revised manuscript we have deleted any reference to the subscales as they were not used and might only add confusion.  We also added several example citations of the GET2 used in other published papers. |
| **Reviewer_1 Comment #15**  Exact p-values should be reported instead of "p < .05". | **Author Response:**  We have corrected this and reported actual p-values. |
| **Reviewer_1 Comment #16**  Figure 1: What types of error bars are displayed here? | **Author Response:**  The error bars were used to represent standard deviations. However, we have opted to use line graphs because they offer a better visual illustration of pre to posttest changes in life skills and entrepreneurial mindset between the intervention and control groups in each country. |
| **Reviewer_1 Comment #17**  In general, the results section needs strong improvement. As it is, it is very hard to follow and to determine which results are actually relevant for the research questions. I would suggest streamlining the results section, to focus on the time*group interactions, which are what is actually hypothesized (in my understanding) and also to refrain from reporting non-significant trends. | **Author Response:**  We have made improvements to the results section by focusing on the hypothesized time*group interactions. We think is it is relevant to show changes in the life skills subscales and country differences. We have also refrained from reporting test results for non-significant trends. |
| **Reviewer_1 Comment #18**  There are a lot of tests reported. How are the effects of multiple testing accounted. | **Author Response:**  We scaled down the tests by focusing time*group interactions and using Bonferroni adjustment for multiple comparisons we considered necessary to address the objectives of the study. |
| **Reviewer_1 Comment #19**  The discussion fails to address the finding that the total scores on life skills did not improve. This should be elaborated in more detail. | **Author Response:**  We reported that there were no interaction effects but main effects for time in the total scores for life skills. We have revised the discussion to explain why this. We went further to show that there were, however, variations in these results at country level where Botswana had interaction effects because the control group had a significantly higher mean at pretests while the intervention group had significantly higher changes at posttest compared to the control group. We addressed the potential source of these differences in the discussion. |
| **Reviewer_2 Comment #20**  Reviewer #2: In general, this is an interesting and attractive subject. However, there are a few areas that need redress. Kindly improve on the connect/links between paragraphs and literature. Let it flow. Strengthen the global and local context from the three African countries. Bring out cases in the sport-based youth Development Programs that exhibited success/failure from both global and local perspectives. Try to inform your reader how PYD play an important role in supporting the all-inclusive development of youths. | **Author Response:**  Thank you for this feedback. We have revised the introduction to address these issues: connections/links between paragraphs and literature, gaps in the literature, how this study sought to address those gaps, the relevance of sport-based youth development programs, entrepreneurship education, inclusive approaches and asset based approaches such as the PYD framework. |
| **Reviewer_2 Comment #21**  The author(s) need to support the discussion about the objectives of the paper. The author's discussion, findings and conclusions should answer the key objectives of the study. The objectives appear to be thrown around. Kindly state the objectives clearly and address them in your findings and conclusion. Avoid mixing them. | **Author Response:**  We have revised the study’s objectives and hypotheses to enhance clarity. We also revised the results section to ensure they are aligned to the stated hypotheses or objectives. We reduced the results reported to ensure the focus is on primary hypotheses. |
| **Reviewer_2 Comment #22**  In your discussion, try to inform your reader on how widely you researched the topic on which you're writing. This is very important for the bibliographic studies | **Author Response:**  Thank you for this feedback. We addressed this when we revised the introduction. We lament the dearth of intervention research and overreliance on cross-sectional studies on this topic because this limits causal inference from the findings. Our suggestion for future research also addresses what could be done to build a body of literature on this topic. I little anecdote is that our project team is already expanding research activity to follow-up on these suggestions. |
| **Reviewer_2 Comment #23**  In addition, the authors may wish to link their study with the current need of international development agencies which have refocused their attention on strategies to enhance livelihoods (i.e, the youths in the three countries can improve), including the UN Development Program and World Bank. Indeed, for example the United Nations (2020) emphasizes livelihoods directly in relation to three of its Sustainable Development Goals (SDGs), e.g. including Goal 1: no poverty. | **Author Response:**  We have referred to the need for increasing collaborations with development partners such as the United Nations Development Program and the World Bank to enhance livelihoods and sustainable development. |
| **Reviewer_2 Comment #24**  On line 58; Write PYD in full for the first time, then abbreviate | **Author Response:**  The correction has been made |
| **Reviewer_2 Comment #25**  107; The author may need have a diagram illustrating the Conceptual Framework in which the relevant study variables are clearly shown. Which data are you dealing with??? If you don’t have to show your conceptual framework, then use econometric models if possible. | **Author Response:**  We have developed a diagram to illustrate the conceptual framework. |
| **Reviewer_2 Comment #26**  359; The authors may delete "test was" | **Author Response:**  Deleted. |
| **Reviewer_2 Comment #27**  The authors may check for more spelling error and spacing. there are many | We have reviewed the manuscript as well as run spell and grammar check to identify locate and correct errors. |
