# Supplementary material for: Effects of a sport-based positive youth development program on youth life skills and entrepreneurial mindsets
Source: PLoS One. 2022 Feb 4;17(2):e0261809. doi: 10.1371/journal.pone.0261809 (PMC8815907; doi:10.1371/journal.pone.0261809)
Supplement: Attachment — Submitted filename: Response to Reviewers 12 9 21.docx [file pone.0261809.s004.docx]

**Manuscript title**: Effects of a Sport-Based Positive Youth Development Program on Youth Life skills and

Entrepreneurial Mindsets

Response to Reviewers

| **Comments** | **Authors’ response** |
| --- | --- |
| Please find below our response to reviewers’ comments. We appreciate the helpful feedback. | |
| **Reviewer_1’s General Comments**  I want to congratulate the authors on a thorough revision of the manuscript. I think that the changes made helped strengthen the contribution of the manuscript. I only have a few minor suggestions to make: | **Authors’ Response:**  Thanks for the positive feedback |
| **Reviewer_1 Technical Comment #1**  Data availability: If I didn’t miss anything, you state that the data set is available but not where it can be accessed. | **Authors’ Response**:  Apologies for not sharing the link. It is: https://doi.org/10.6084/m9.figshare.17125589 |
| **Reviewer_1 Technical Comment #2**  Power: Thank you for adding a power analysis. However, a lot of your discussion focuses on differences between the three samples – something that you did not plan for in your power analysis. I would suggest to substantially shorten the discussion of the differences between the three samples as the sample sizes in each country do not seem sufficient for drawing meaningful conclusions on this question | **Authors’ Response**:  Your concern about power analyses is appreciated. We have added text to the discussion on country comparisons, to explain how sample size limitations may limit generalizability of findings beyond the current sample.  We note that Power calculations are used as a tool to help researchers when designing studies and determining sample sizes needed to avoid making a Type 2 error (i.e., the intervention had an effect, but the sample size was too small to detect it). In our case the intervention had effects that we believe are worth reporting for the following reasons:   1) This study used a fairly novel and exploratory approach with samples drawn from three countries to test the utility of a sport-base PYD intervention. Because of this there was no good way to determine the effect size needed for power calculations. This is why we used Cohen’s d, for power calculation because it is recommended when a literature review or pilot study cannot inform effect size (Cohen, 1988). Power calculations are recognized as offering little value to early exploratory studies (such as this) where scarce data is available on which to make power calculations (Jones & Sommerlund, 2007). 2) The intervention described in this program was resource intensive as it involved 14-day and 8-day overnight camp experiences in three different countries. This meant the sample size of around 50 in each country was determined to be reasonable to detect the expected effects from the intervention.   Regardless of the appropriateness of power calculations for this particular study, our discussion focuses only on statistically significant results. This means the sample size we used was large enough to detect at least some effects. It’s certainly possible that other effects might have been detectable with larger sample sizes in each country, however we did not bring such possibilities into the discussion. Shortening the discussion of the statistically significant findings on country difference will lead to a missed opportunity to report on these important findings. |
| **Reviewer_1 Technical Comment #3**  A minor thing but I thought it was confusing that the samples a referred to by the country name. I would suggested referring to the „Ghanaian sample“ or „ sample collected in Ghana“ instead of just „Ghana“, for instance. | **Authors’ Response**:  Thank you for this feedback. We have made appropriate changes. |
| **Reviewer_2 General Comments:** The current text of authors' manuscript suggests that all comments highlighted in the earlier review have been satisfactorily addressed. The manuscript is technically sound in its current state as data appear to support the conclusions. I am certainly convinced that this manuscript is now fit for publication by this journal. | **Authors’ Response**:  Thank you for this positive feedback |

**References**

Cohen, J. (1988). *Statistical Power Analysis for the Behavioral Sciences* (2nd ed.). Hillside, NJ: Lawrence Erlbaum Associates.

Jones, A., & Sommerlund, B. (2007). A critical discussion of null hypothesis significance testing and statistical power analysis within psychological research. Nordic Psychology, 59(3), 223–230. [https://doi.org/10.1027/1901-2276.59.3.223](https://psycnet.apa.org/doi/10.1027/1901-2276.59.3.223)
